# Multiple Imputation to Correct for Nonresponse Bias: Application in Non-Communicable Disease Risk Factors Survey

**DOI:** 10.5539/gjhs.v8n1p133

**Published:** 2015-05-15

**Authors:** Hamid Heidarian Miri, Jafar Hassanzadeh, Abdolreza Rajaeefard, Majid Mirmohammadkhani, Kambiz Ahmadi Angali

**Affiliations:** 1 Department of Epidemiology, School of Health, Shiraz University of Medical Sciences, Shiraz, Iran; 2 Department of Epidemiology, Research Center for Health Sciences, School of Health, Shiraz University of Medical Sciences, Shiraz, Iran; 3 Research Center for Social Determinants of Health, Department of Community Medicine, School of Medicine, Semnan University of Medical Sciences, Semnan, Iran; 4 Biostatistics Department, Health Faculty, Ahvaz Jundishapur University of Medical Sciences, Ahvaz, Iran

**Keywords:** survey, multiple imputation, nonresponse bias

## Abstract

**Background:**

This study was carried out to use multiple imputation (MI) in order to correct for the potential nonresponse bias in measurements related to variable fasting blood glucose (FBS) in non-communicable disease risk factors survey conducted in Iran in 2007.

**Methods:**

Five multiple imputation methods as bootstrap expectation maximization, multivariate normal regression, univariate linear regression, MI by chained equation, and predictive mean matching were applied to impute variable fasting blood sugar. To make FBS consistent with normality assumption natural logarithm (Ln) and Box-Cox (BC) transformations were used prior to imputation. Measurements from which we intended to remove nonresponse bias included mean of FBS and percentage of those with high FBS.

**Results:**

For mean of FBS results didn’t considerably change after applying MI methods. Regarding the prevalence of high blood sugar all methods on original scale tended to increase the estimates except for predictive mean matching that along with all methods on BC or Ln transformed data didn’t change the results.

**Conclusions:**

FBS-related measurements didn’t change after applying different MI methods. It seems that nonresponse bias was not an important challenge regarding these measurements. However use of MI methods resulted in more efficient estimations. Further studies are encouraged on accuracy of MI methods in these settings.

## 1. Introduction

According to the fact sheets of World Health Organization, updated in January 2015, diabetes account for 1.5 million deaths annually while more than 80% of these deaths happen in low- and middle-income countries (World Health Organization [WHO], 2014).

Provision of accurate information on prevalence of diabetes and its major risk factors will enable us to understand the importance of burden which it imposes on societies and can also be regarded as a necessary part to devise and monitor preventive and controlling programs.

One of the best tools to reach this purpose is STEPS study, devised by WHO, which is a nationwide population based cross-sectional survey to gather information on non-communicable disease risk factors as well as diabetes.

One important issue about STEPS studies is that usually a fraction of sample don’t take part in the whole study or some part of it known as nonresponse that can threaten the results of the study by reducing the sample size and in some cases biasing descriptive and analytic statistics.

For example there have been about 17% nonresponses in Fasting Blood Glucose measurement in almost all rounds of these surveys in Iran while no advanced strategies has been applied to treat possible nonresponse bias (Asgari, Mirzazadeh, & Heidarian, 2009; Esteghamati et al., 2008).

There are a wide range of methods to deal with nonresponses (Little & Rubin, 1989). The most approved method is multiple imputation (MI) proposed by Rubin which has now stablished its status in many area of research (Sterne et al., 2009).

The present study aimed to use MI method to correct for potential bias in FBS-related measurements due to nonresponses in Iran’s latest published STEPS study conducted by Iran’s Ministry of Health in 2007.

Five imputation methods were applied to assess the sensitivity of results to the imputation approach and two transformations were used to make variable FBS consistent with normality assumption.

## 2. Methods

### 2.1 Subjects and Sample Size

We used data from Iran’ STEPS study conducted in 2007 in which 30000 of Iranian people aged 15 to 64 years had been recruited. Data were collected through a questionnaire and by taking a fasting blood sample. We excluded pregnant women and also people aged under 25 since they were not subjected to the biochemical measurements. Finally a data set of 23663 observations and 39 variables was used for the present study. Although detailed information on this survey and its results can be found elsewhere (Asgari, Aghajani, Haghazali, & Heidarian, 2009; Asgari, Mirzazadeh, et al., 2009), some related aspects are described in the following sections.

### 2.2 Survey Setting

To fully grasp survey setting, it is helpful to get familiar with the sampling frame. As it was intended to have survey results represent the whole country in age group 15 to 64 years, determined sample were equally distributed over all 30 provinces, 5 ten-year age groups, and 2 sexes. So 100 people for each sex-age group were selected from each province.

To select 1000 people in each province 50 households were randomly chosen using postal codes. Each selected household and its neighborhood constituted a cluster. Then starting from chosen household, data collectors proceeded in a pre-specified direction to fill in the study questionnaire for 2 individuals in each sex-age group (20 in each cluster). Provinces and clusters were then used respectively for strata and sampling unit in the survey setting. Linearized was set as the type of standard error. Survey setting and weights used in this study was the same as defined by survey team.

### 2.3 Survey Weights

For each sex-age group-province stratum, base weight was its respective number of people in the target population. To account for differences in the size of strata due to nonresponses or possible errors, the adjusted weights were defined as base weights divided by the existing size of the strata. This approach of adjusting weights was also seen in other studies (Schenker et al., 2006; Taylor et al., 2002).

### 2.4 Nonresponse Rate

In the dataset we received the rate of missing data ranged from about 0.01% to 17.80%. The target variable, FBS, contained 17.47% missing values. Only 35.8% of variables were fully observed and 69.2% of observation had all variables observed.

### 2.5 Mechanism of Missing Data

There are three mechanisms responsible for missing data: 1) missing completely at random under which the probability of being missing depends neither to the values of other variables nor to the values of missing information, 2) missing at random in which probability of being missing depends to other variables but conditioning on which is independent from the value of missing information, 3) missing not at random that the likelihood of being missing for a given value depends to its value after conditioning on other variables (Little & Rubin, 2002; Rubin, 1976, 1987). With real data sets in which true values of missing data are unknown and all variables related to missingness are not captured it is impossible to prove which mechanism has produced missing data (Joseph L. Schafer, & Olsen, 1998). So we proceeded with no assumption about missing data mechanism.

### 2.6 MI Procedures

MI, proposed by Rubin in 1987 (Rubin, 1987), substitutes each missing value with two or more (M) values and produces M data sets that are complete regarding the imputed variables. Then each data set is analyzed separately using standard statistical analysis and finally these results are combined according to Rubin’s combination rule defined in formulas 1 to 4 (Rubin, 1987). There can be found a wide range of methods and softwares (Horton & Lipsitz, 2001) for proper (Rubin, 1987) multiple imputation.


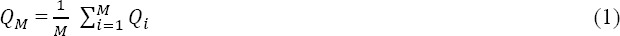



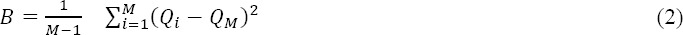



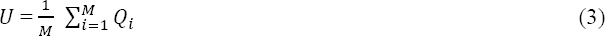



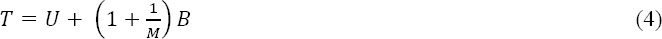


*Q_M_: overall point estimate, Q_i_: point estimate of i-th imputed data set, M: number of imputation, B: between imputation variance, U: within imputation variance, T: total variance*.

In this study five MI methods were selected as: 1) bootstrap expectation maximization algorithm (BEM) which uses expectation maximization algorithm introduced by Dempster (Dempster, Laird, & Rubin, 1977; Joseph L. Schafer, 1997) to estimate the posterior distribution of incomplete data and then a bootstrap approach to take draws from this posterior distribution (Honaker, King, & Blackwell, 2011). In this study BEM algorithm was performed by R based AMELIA II package version 1.7.2. As this program allows to simultaneously impute more than one variables without discrimination between dependent and independent variables, all biochemical variables were included in the imputation process too. Imputed data set was saved in Stata 11 format for subsequent analysis, 2) multiple imputation by chained equation (MICE), also known as switching regression or sequential regression multivariate imputation (Kennickell, 1991), is based on fully conditional specification models. It doesn’t depend on multivariate normality assumption (Raghunathan, Lepkowski, Van Hoewyk, & Solenberger, 2001) and can impute any type of variables (White, Royston, & Wood, 2011). Here we used Stata implementation of this procedure through ice command with the default number of 10 cycles (StataCorp. 2009. Stata 11 Multiple-Imputation Reference Manual. College Station). Like BEM, biochemical variables were included in the imputation process. Information on how to install and use MICE program can be found elsewhere (Royston & White, 2011), 3) multivariate normal regression (MVN) with which missing values are imputed by taking values from posterior distribution estimated by Markov Chain Monte Carlo algorithm (Lee & Carlin, 2010; Joseph L. Schafer, 1997). It is based on the multivariate normality assumption but will give valid results even when this assumption, like this study, is violated (Lee & Carlin, 2010; Joseph L. Schafer, 1997). With MVN it is possible to define several response variables but predictors should be completely observed. For MVN and two next methods we used MI system implemented in Stata version 11.2 (StataCorp. 2009. Stata 11 Multiple-Imputation Reference Manual. College Station) and biochemical variables were excluded from imputation process, 4) univariate linear regression (UVR) which uses a posterior predictive distribution derived from a normal linear univariate regression model. The imputation model comprises an imputing variable as a response and a number of predictors with no missing value (StataCorp. 2009. Stata 11 Multiple-Imputation Reference Manual. College Station), and 5) predictive mean matching (PMM) that is a semi-parametric version of the UVR with the same procedure but instead of replacing missing values with what the model predicts, PMM takes draws randomly from a set of observed values whose predictive means are in a pre-specified distance of that of missing values (StataCorp. 2009. Stata 11 Multiple-Imputation Reference Manual. College Station).

Although some experts advised that unless nonresponse rate is not unusually high more than five to ten imputation has no additional gain in efficiency (Rubin, 1987; J. L. Schafer, 1999), in complex surveys more number of imputation is encouraged to yield valid estimates (Wang & Robins, 1998). So the number of imputation was set to 20, as suggested by Graham (2009).

To make the heavily positively skewed FBS consistent with normality assumption natural logarithm (Ln) and Box-Cox (BC) transformations were used prior to imputation. For convenience we used O, Ln, or BC plus the abbreviation for MI methods to refer to the method and the scale of FBS. For example O-PMM refers to predictive mean matching method applied to original scale of FBS.

### 2.7 Variables Used in MI Models

As it is suggested variables correlated with participation or FBS’s values (Horton & Lipsitz, 2001) besides variables associated to sampling and weights (Kim, Michael Brick, Fuller, & Kalton, 2006; Schenker et al., 2006) were used in MI models.

To assess which variable were related to participation (Davey, Shanahan, & Schafer, 2001) one binary indicator variable was made which took 1 for those who were missing for all biochemical measurements and zero for those who had at least one of them observed. Then we used this variable as an outcome in a logistic regression model and all other variables as predictors. Variables with P value less than 0.2 were selected for MI model.

To find out which variables were related to FBS’s values, we fitted a linear regression for FBS on all other variables in the data set. Then *P* value less than 0.2 was used as a criterion for inclusion in MI model. Survey setting was regarded in both logistic and linear regression modes (Schenker et al., 2006).

Totally 23 variables were selected for imputation of FBS as three nominal variables for province, fastening seat belt and wearing crash helmet habits; eight binary variables for residential area, sex, physical activity, currently use of tobacco, history of diabetes in person and their family, taking anti-diabetic medication (two variables); twelve continuous variables for age, sedentary life style, height, weight, waist circumference, systolic and diastolic blood pressure, total cholesterol, HDL cholesterol, triglyceride; and two variables for weights and sampling unit.

Twelve of these variables contained some amounts of missing values. Although, except for three biochemical variables, the most amount of missing data was 0.15%, we couldn’t use them in univariate imputation models. To not having to set aside these variables with few amount of missing data, we used single imputation to impute them prior to imputation of FBS. For this purpose MICE method was used and all variables except for biochemical measurements that had large amount of missing data were included in the imputation models. The imputed data set was used for imputation of FBS.

### 2.8 Statistical Analysis

Statistical analysis falls into two categories: first, measurements related to FBS from which we intended to remove nonresponse bias including: 1) Mean of fasting blood sugar, 2) proportion with FBS equal to or greater than 110 mg/dl but less than 126 mg/dl called impaired fasting sugar (IFG), 3) proportion with FBS equal to or greater than 126 mg/dl called high blood glucose (HBG). Those taking anti-diabetic medication were excluded from calculations of mean.

Second, measurements for evaluation of MI’s as: relative variance increased (RVI), fraction of missing information (FMI), and relative efficiency (RE) defined in formulas 5 to 8 (Rubin, 1987; Joseph L. Schafer, 1997; StataCorp. 2009. Stata 11 Multiple-Imputation Reference Manual. College Station):


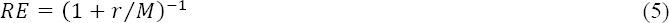



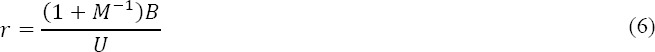



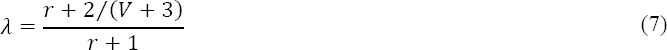



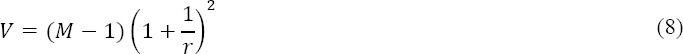


*RE: relative efficiency, r: relative variance increased, λ: fraction of missing information, V: degrees of freedom, M: number of imputation, B: between variance imputation, U: within variance imputation*.

All calculations were performed using Stata’s “mi estimate: svy linearized” command in which analysis and combination steps were integrated and survey setting is taken in to account.

## 3. Results

Table 1 through Table 3 display point estimates with 95% CI, estimated standard error, RE, FMI, and RVI for mean of FBS, prevalence of IFG and prevalence of HBG respectively for available case analysis (AC), adjusting weights (AW), and imputation strategies. The results from AW and AC were almost the same for these easurements.

**Table 1:** Point Estimates of Mean of Fasting Blood Sugar with 95% CI, Estimated Standard Errors, Relative Efficiency, Fraction of Missing information, and Relative Variance Increase for Multiple Imputation Methods and Transformations, STEPS Study, Iran 2007

Transformation	Imputation Method	Mean	95% CI	SE	RE	FMI	RVI
No	AC^a^	89.45	(88.87,90.02)	0.2922			
	AW^b^	89.19	(88.59,89.79)	0.3061			
	MICE^c^	89.41	(88.62,90.2)	0.3976	0.9791	0.4270	0.7141
	MVN^d^	89.35	(88.63,90.08)	0.3678	0.9823	0.3607	0.5443
	UVR^e^	89.26	(88.57,89.95)	0.3484	0.9852	0.3004	0.4165
	PMM^f^	89.37	(88.75,89.99)	0.3142	0.9863	0.2781	0.3745
	BEM^g^	89.68	(88.95,90.41)	0.3688	0.9811	0.3862	0.6053
Natural Logarithm	MICE	89.42	(88.82,90.03)	0.3085	0.9865	0.2731	0.3655
	MVN	89.34	(88.74,89.94)	0.3048	0.9856	0.2924	0.4012
	UVR	89.36	(88.8,89.91)	0.2812	0.9913	0.1749	0.2083
	PMM	89.31	(88.71,89.9)	0.2998	0.9876	0.2520	0.3284
	BEM	89.34	(88.76,89.92)	0.2950	0.9878	0.2466	0.3191
Box Cox	MICE	89.30	(88.71,89.88)	0.2951	0.9868	0.2681	0.3566
	MVN	89.21	(88.69,89.72)	0.2631	0.9953	0.0950	0.1040
	UVR	89.23	(88.7,89.76)	0.2702	0.9919	0.1623	0.1906
	PMM	89.34	(88.66,90.03)	0.3471	0.9805	0.3974	0.6338
	BEM	89.14	(88.58,89.7)	0.2835	0.9879	0.2460	0.3181

a Available case analysis;

b Adjusting weights;

c Multiple imputation by chained equations;

d Multivariate normal regression;

e Univariate regression;

f Predictive mean matching;

g Bootstrap expectation maximization algorithm.

**Table 2:** Point Estimates of prevalence of IFG^a^ with 95% CI, Estimated Standard Errors, Relative Efficiency, Fraction of Missing information, and Relative Variance Increase for Multiple Imputation Methods and Transformations, STEPS Study, Iran 2007

Transformation	Imputation Method	Percentage	95% CI	SE	RE	FMI	RVI
No	AC^b^	3.43	(3.07,3.43)	0.0017			
	AW^c^	3.35	(2.96,3.35)	0.0020			
	MICE^d^	5.44	(4.75,5.44)	0.0035	0.978655	0.43622	0.740867
	MVN^e^	5.49	(4.83,5.49)	0.0033	0.979827	0.411762	0.671815
	UVR^f^	5.34	(4.7,5.34)	0.0032	0.980873	0.389996	0.614876
	PMM^g^	3.36	(2.91,3.36)	0.0022	0.978231	0.445068	0.7673
	BEMh	5.47	(4.79,5.47)	0.0035	0.979293	0.422888	0.702523
Natural Logarithm	MICE	4.89	(4.2,4.89)	0.0035	0.972627	0.562863	1.218282
	MVN	4.85	(4.29,4.85)	0.0028	0.982666	0.352806	0.526155
	UVR	4.81	(4.23,4.81)	0.0030	0.978368	0.442207	0.758664
	PMM	3.5	(3.11,3.5)	0.0020	0.987557	0.252005	0.328389
	BEM	4.85	(4.26,4.85)	0.0030	0.978945	0.430155	0.723208
Box Cox	MICE	4.18	(3.62,4.18)	0.0028	0.977899	0.452021	0.788654
	MVN	4.08	(3.63,4.08)	0.0022	0.985906	0.285914	0.388972
	UVR	4.09	(3.59,4.09)	0.0024	0.979456	0.419498	0.693046
	PMM	3.32	(2.93,3.32)	0.0020	0.98475	0.309733	0.434906
	BEM	4.03	(3.59,4.03)	0.0022	0.987151	0.260333	0.34278

a Fasting blood glucose equal to or greater than 110 mg/dl but less than 126 mg/dl

b Available case analysis

c Adjusting weights

d Multiple imputation by chained equations

e Multivariate normal regression

f Univariate regression

g Predictive mean matching h.Bootstrap expectation maximization algorithm

**Table 3:** Point Estimates of prevalence of HBG^a^ with 95% CI, Estimated Standard Errors, Relative Efficiency, Fraction of Missing information, and Relative Variance Increase for Multiple Imputation Methods and Transformations, STEPS Study, Iran 2007

Transformation	Imputation Method	Percentage	95% CI	SE	RE	FMI	RVI
No	AC^b^	5.56	(5.08,6.05)	0.0024			
	AW^c^	5.45	(4.92,5.98)	0.0026			
	MICE^d^	7.35	(6.63,8.07)	0.0036	0.9841	0.3229	0.4617
	MVN^e^	7.27	(6.57,7.97)	0.0036	0.9833	0.3397	0.4972
	UVR^f^	7.29	(6.63,7.96)	0.0035	0.9865	0.2731	0.3654
	PMM^g^	5.47	(4.99,5.94)	0.0024	0.9927	0.1462	0.1687
	BEM^h^	7.4	(6.56,8.24)	0.0042	0.9749	0.5144	1.0068
Natural Logarithm	MICE	5.71	(5.19,6.23)	0.0026	0.9901	0.2004	0.2456
	MVN	5.63	(5.11,6.14)	0.0026	0.9884	0.2350	0.3000
	UVR	5.66	(5.16,6.17)	0.0024	0.9913	0.1751	0.2085
	PMM	5.32	(4.82,5.82)	0.0024	0.9880	0.2431	0.3133
	BEM	5.63	(5.12,6.14)	0.0026	0.9888	0.2257	0.2848
Box Cox	MICE	5.44	(4.94,5.94)	0.0024	0.9893	0.2157	0.2691
	MVN	5.38	(4.9,5.86)	0.0024	0.9906	0.1898	0.2298
	UVR	5.4	(4.92,5.89)	0.0024	0.9907	0.1873	0.2261
	PMM	5.45	(4.98,5.92)	0.0024	0.9919	0.1642	0.1931
	BEM	5.34	(4.86,5.82)	0.0024	0.9889	0.2240	0.2821

a Fasting blood glucose equal to or greater than 126 mg/dl;

b Available case analysis;

c Adjusting weights;

d Multiple imputation by chained equations;

e Multivariate normal regression;

f Univariate regression;

g Predictive mean matching;

h Bootstrap expectation maximization algorithm.

Table 1 shows that using MI and transformations didn’t significantly change the mean of FBS. While BC-MVN had the highest relative efficiency and lowest fraction of missing information and relative variance increase, O-MICE had the lowest relative efficiency and highest fraction of missing information and relative variance increase.

The ratio of standard error before imputation to standard error after it is another important metric that is expected to be more than one meaning that use of MI results in smaller standard error. This ratio exceeded one only for BC-MVN, BC-BEM, BC-UVR, and Ln-UVR.

Table 2 indicates that prevalence of IFG became significantly higher after applying all imputation methods except for PMM which gave similar results to those of AC. Estimation of prevalence of IFG was highly influenced by transformations as using BC transformation resulted in a reduction in it for all imputation methods and using natural logarithm did so for just UVR. Regarding RE, FMI, and RVI; Ln-PMM and Ln-MICE had the best and worst performance respectively. All MI models had the ratio of standard error before imputation to standard error after it less than one that was not a desired quality.

Table 3 shows that all MI methods, except for PMM, tended to increase the prevalence of HBG. The impact of transformation was more noticeable here as both transformations caused a decrease in the results. O-PMM had the highest relative efficiency and lowest fraction of missing information and relative variance increase while O-BEM had the lowest relative efficiency and highest fraction of missing information and relative variance increase. LN-UVR, Ln-PMM and Box-Cox transformation with all MI models had the ratio of standard error before imputation to standard error after it equal to one.

## 4. Discussion

In this study we applied five imputation strategies across two transformations to impute variable FBS in NCD’s risk factor survey data in order to correct for potential nonresponse bias in estimation of FBS-related measurements. The choice of MI methods was based on type and distribution of imputing variable, subsequent analyses, and software considerations.

The results from MI’s were compared with those of available case analysis and adjusting weights methods which were of similar results as was the case in another study that had the same approach for adjusting weight as we did. They reasoned that this similarity is because of the fact that few variables were used to define strata (Schenker et al., 2006).

There was also no significant difference among the results of different imputation models that was in line with other works (Lee & Carlin, 2010; Lin, 2010; Taylor et al., 2002). Surprisingly we also found no significant difference in the mean of FBS after applying imputation models. Considering the fact that there were some variables related to both participation and FBS’s values we would expect that the study results could have suffered from a kind of selection bias. The justification could be that the selection bias in FBS could have been compensated by the presence of some variables related to participation in the same direction and related to FBS in the opposite directions and vice versa. So if there was one variable that caused an increase in the mean of FBS by increasing nonresponse probability there was another variable that caused a decrease in the mean of FBS by increasing nonresponse probability.

Considering prevalence of IFG and HBG, we saw a similarity between PMM and AC which can be explained by PMM strategy for replacing missing values which produces a distribution similar to that of observed values on the part of missing data. Other MI methods tended to increase regarding measurements. Performances of MI’s in estimations of prevalence of IFG and HBG were highly influenced by transformations as they, especially Box-Cox, pull the right tail of distribution towards the mean and therefore the imputed values are placed nearer to the mean rather than far from it. This is why the estimated prevalence of HBG is more affected by transformation because, in comparison to IFG, further part of the distribution contributes to it. So as it is said that PMM is a reliable approach when data is heavily skewed and measurement of interest is proportion (Lee & Carlin, 2010) we concluded that there was no serious bias in the estimation of prevalence of IFG and HBG in the regarding data set. However PMM method on Ln transformed FBS produced more efficient estimates.

The ratio of standard error before imputation to standard error after it was less than one for some models meaning that they tended to increase the variance. The large variance of imputed FBS could be explained by low correlation among variables used in MI models. It has been shown that variance of MI will be higher when the correlation among variables is less than 0.28 and there is a high rate of missing data (Gómez-Carracedo, Andrade, López-Mahía, Muniategui, & Prada, 2014). The impact of clustering is also important in increasing variance. Taking the mean of FBS, ratio of standard error of O-MICE to that of BC-MVN was 0.79 but with taking account of sampling frame became 1.06. Ratio of standard error with sampling taken into account to standard error without it was 1.79 for O-MICE but 1.33 for BC-MVN. So it seems that design effect is higher for O-MICE than for BC-MVN resulting in larger variance.

Regarding quantities used for evaluation of imputation models, BC-MVN was among top five imputation models for all measurements implying that MVN on Box-Cox transformed data outperformed other approaches (Lee & Carlin, 2010). Besides FMI was substantially less than nonresponse rate in case of BC-MVN indicating this approach was highly predictive (Lewis et al., 2014). The superior performance of MVN might not be attributed to more number of variables in MI models since the same variables were used in MICE too. In addition it is shown that the number of variables has little effect on the performance of MI especially when the correlation among them is low (Lin, 2010) like what we saw in this study.

The advantage of present study was the use of multiple imputation in STEPS data in order to remove the serious doubt which nonresponses would have cast on FBS-related measurements. We applied a wide range of methods to assess the sensitivity of results to the imputation strategy.

The main limitation of present study was that true values of missing information were unknown so we couldn’t assess the accuracy of MI models and as a results the finding of present study couldn’t be generalized to other biochemical measurements or surveys of other years. There also was no information of unit nonresponse (those who refused to take part in the whole survey) rate and the argument that all selected people has agreed to take part in the first two steps because information was collected by interview needs to be put in perspective as it was seen in Flint Men’s Health Study that response rate was 86% for interview step (Taylor et al., 2002). We suggest that at least some useful information be recorded for nonresponses like sex, approximate age, district, and so on. We also had no additional information about item nonresponses (those who refused to take part in a part of survey) like the number of attempts made for invitation. In a study that used MI to correct for nonresponse bias the number of invitations was incorporated in MI process to improve the results (Kmetic, Joseph, Berger, & Tenenhouse, 2002).

## 5. Conclusion

Application of multiple imputation methods didn’t change the FBS-related finding of NCD risk factors survey conducted in Iran in 2007 so it is concluded that nonresponse bias was not an important challenge regarding these measurements. However the benefit of applying MI method, especially MVN, in this setting was reaching more efficient estimations by retrieving some of missing information. Further studies are needed to assess the accuracy of MI approaches and also on how to improve MI performance in similar settings.
